# Polyphenylene Sulfide-Based Compositions with Solid Fillers for Powder Injection Molding

**DOI:** 10.3390/polym18030341

**Published:** 2026-01-28

**Authors:** Dmitry V. Dudka, Azamat L. Slonov, Khasan V. Musov, Aslanbek F. Tlupov, Azamat A. Zhansitov, Svetlana Yu. Khashirova, Alexander Ya. Malkin

**Affiliations:** 1 A.V. Topchiev Institute of Petrochemical Synthesis, Russian Academy of Sciences (TIPS RAS), 29 Leninsky Prospekt, 119991 Moscow, Russia; dudka@ips.ac.ru; 2 Center for Progressive Materials and Additive Technologies, H.M. Berbekov Kabardino-Balkarian State University, st. Chernyshevsky, 173, 360004 Nalchik, Russia; azamatslonov@yandex.ru (A.L.S.); xmusov@gmail.com (K.V.M.); tlupovaslanbek99@gmail.com (A.F.T.); azamat-z@mail.ru (A.A.Z.); new_kompozit@mail.ru (S.Y.K.)

**Keywords:** powder molding, viscosity, visco-elastic properties, polyphenylene sulfide, suspensions

## Abstract

Powder Injection Molding (PIM) is a versatile manufacturing technology widely used for fabricating components with complex geometries from metals and ceramics, yet its application to high-performance thermoplastics remains underutilized. This study explores the feasibility of manufacturing products from Polyphenylene Sulfide (PPS)—a promising linear aromatic polymer synthesized in powder form—using PIM technology and investigates the development of PE-based feedstocks with PPS and solid fillers. Regarding the matrix formulation, it was found that using pure paraffin as a binder limited the maximum PPS content to 20%. Consequently, a modified binder system consisting of Low-Density Polyethylene (LDPE) and paraffin in a 70:30 wt.% ratio was utilized, which successfully increased the PPS loading in the feedstock to 50% and enabled stable molding. Following matrix optimization, the study examined composites incorporating various fillers, including chalk, talc, and carbon fibers. Systematic rheological analysis confirmed that these composite suspensions possess characteristics necessary for molding products with complex geometries. Key results indicate that optimal sintering conditions were established to achieve the required mechanical properties. Among the tested fillers, carbon fibers were the most effective reinforcement, increasing the elastic modulus by 33% and flexural strength by 20%. Representative examples of samples successfully manufactured via this approach are presented.

## 1. Introduction

Powder Injection Molding technology (PIM) is attracting significant interest due to its cost-effectiveness, particularly in the production of small items with complex geometries, and the ability to produce functional parts with minimal raw material loss. This method involves forming parts from highly filled suspensions (feedstocks) consisting of a binder and dispersed particles of the base material. It is widely used in the processing of metal [1,2,3,4] and ceramic powders [5,6,7], followed by binder removal and sintering under pressure.

A promising direction is the use of high-temperature polymer powders in PIM technology, which are difficult to process using traditional methods, as was shown in the example of Polyetheretherketone (PEEK) and Polyphenylene Sulfone (PPSU) [8,9,10]. These materials also include other engineering plastics, particularly Polyphenylene Sulfide (PPS), which have high melting or flow points, making their processing by standard methods difficult or ineffective, especially if there is a need to manufacture a small series of products with a complex geometric configuration. It should be noted that powder casting technology is a relatively new area of polymer processing, and publications on this topic are very limited.

PIM technology includes four main stages [11]: obtaining a highly filled suspension (also called feedstock) based on a binder and functional powder material; injection molding (obtaining a “green” part); removing the binder (debinding) under the influence of temperature or chemical reagents (obtaining a “brown” part); sintering the powder filler at the appropriate temperature and obtaining the final product. This sequence of operations also applies to powder molding of polymers.

This paper continues our research into the powder casting of engineering polymers, previously conducted for PEEK and PPSU. As in previous cases, this study will include a consideration of the rheological properties of the composites and powder sintering conditions. This paper focuses on PPS, a polymer that has recently entered technical practice [12,13,14,15,16]. Compared to other high-temperature engineering plastics, PPS exhibits increased rigidity, wear resistance, resistance to impact loads and crack formation at temperatures up to 220 °C (short-term up to 270 °C), fire resistance, and resistance to aggressive environments [17,18].

## 2. Materials and Methods

### 2.1. Materials

**Figure 1:**
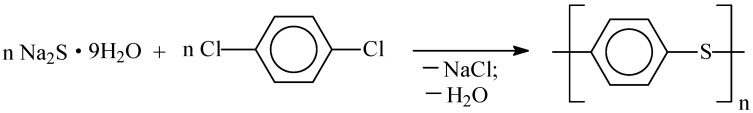
Scheme of PPS synthesis.

The object of study was PPS produced by Scientific and Technical Center Akhmadullini Ltd. (Kazan, Russia). The polymer was synthesized by high-temperature polycondensation via a two-stage nucleophilic substitution mechanism in a mixture of N-methylpyrrolidone and toluene (Figure 1) [12,19]. Lithium acetate and/or lithium oxalate were used as catalysts. In the first stage, the crystal hydrate water contained in the Na_2_S hydrate was distilled off. After complete removal of water, until an azeotropic solution was obtained, 1,4-dichlorobenzene was added to the reaction mixture, and the synthesis was carried out at 235–265 °C.

The polymer was characterized by the Melt Flow Index (MFI), which was determined using a capillary viscometer according to ASTM-1238 [20] at a temperature of 350 °C and a load of 50 N. The melt index of the sample used in the work was equal to 200 g/10 min.

The following fillers were used: Nigtalk 2x talc (Nigtas Anonim Sirketi, Simferopol, Russia), with an average particle size of 4 µm; ground carbon fibers from R&G (Waldenbuch, Germany) with an average length of 0.2 mm; calcium carbonate (chalk) grade M-1, produced by Industrial Fillers Ltd. (Moscow, Russia); nanosized pyrogenic silicon dioxide grade A300 (Aerosil, Evonik Industries AG, Essen, Germany), i.e., with a specific surface area of approximately 300 m^2^/g. The mixing of the PPS powder with fillers was carried out on a VLM-40high-speed mixer from Vilitek LLC (Moscow, Russia), with a maximum rotation speed of up to ~2618 rad/s.

However, talc mineral deposits are known to contain amphibole asbestos contamination (tremolite, anthophyllite), with Food and Drug Administration (FDA) testing documenting 17% contamination rates in sampled commercial products [21]. Additionally, occupational inhalation exposure to talc has been associated with pulmonary talcosis, respiratory disease, and mesothelioma risk [22,23]. Given these documented health concerns, the use of talc in this initial proof-of-concept study is justified solely for establishing the viability of the LDPE/paraffin binder system and its compatibility with PPS reinforcement.

The following were used as binders for the preparation of suspensions: food-grade highly purified paraffin grade P-2—a solid translucent white substance, tasteless and odorless, with a melting point of ~52 °C—and Low-Density Polyethylene (LDPE) grade LDPE 15803-020-S-P (OOO Gazprom Neftekhim Salavat, Salavat, Russia)—a granular material with a density of 0.919 g/cm^3^ and a melt flow index of 2.0 g/10 min.

The experimental compositions were selected based on the peer-reviewed literature demonstrating optimal reinforcement at specified loading levels. Aerosil at 1 wt% aligns with works in the literature showing that peak nanosilica effects occur at 0.2–1.0 wt% in PPS nanocomposites [24]. Talc at 5 wt% operates in the linear strengthening regime, significantly below the 30 wt% saturation point where property enhancement plateaus [25]. Carbon Fiber at 10 wt.% represents the practical processing optimum for Powder Injection Molding, where the literature documents substantial mechanical improvements (>100% tensile strength increase) while maintaining adequate mold-filling characteristics [26,27]. The presence of talc and calcium carbonate at 10 wt% follows established practice for particulate reinforcement in thermoplastics, providing effective stiffness enhancement without excessive viscosity increases that compromise PIM processability [25].

### 2.2. Rheological Measurements and SEM Analysis

Rheological properties were studied using a Dynisco LCR-7001 capillary rheometer (Franklin, SA, USA) and an RS-600 rotational rheometer (ThermoHaake, Karlsruhe, Germany) at various shear rates and angular velocities, respectively. Microscopic studies were carried out on a Vega 3 Scanning Electron Microscope (SEM) from Tescan (Brno, Czech Republic). Imaging was conducted at a 5 kV accelerating voltage and $2\times 10^{-2}$ Pa pressure with a working distance of 10 mm. A Secondary Electron (SE) detector was employed to assess surface morphology. A minimum of 5 specimens were examined, with 3 regions per specimen imaged.

### 2.3. Processing

Injection molding of the samples was performed on an SZS-20 injection molding machine (Haitai Machinery, Ningbo, China). PE-containing filaments were produced using a TwinScrew 10 benchtop twin-screw micro extruder with L/D = 30 (TwinTech, Stoke on Trent, UK) at the following temperature zones: T_1_ = 105 °C, T_2_ = 125 °C, T_3_ = 140 °C, T_4_ = 150 °C, and T_5_ = 140 °C.

**Figure 2:**
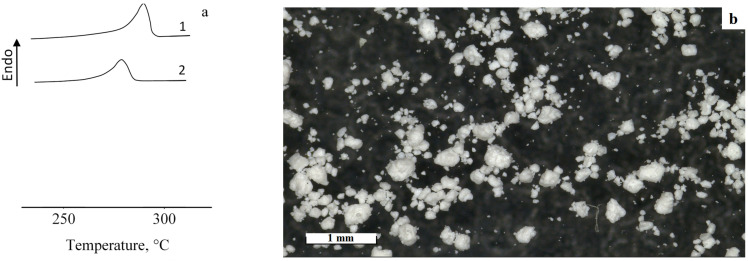
Characteristics of PPS: (**a**) DSC curve of PPS (1—first heating; 2—second heating); (**b**) microphotograph of PPS.

Sintering of the PPS powder was carried out in a temperature range from 270 to 320 °C in 10 °C increments for 1 h. The temperatures were selected based on the thermal properties of the PPS. According to Differential Scanning Calorimetry DSC data (Figure 2). The endothermic peak is observed in the range from 260 to 310 °C. The melting point during the first heating is 300 °C, while during reheating, the temperature decreases to 279 °C.

**Figure 3:**
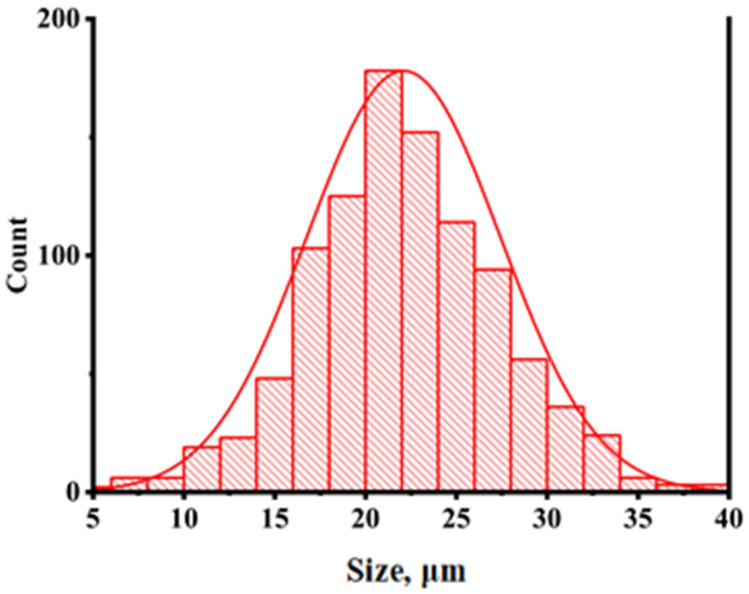
Particle size distribution of PPS powder.

The powder particles are fairly large agglomerates, reaching up to 300 µm in size. To characterize particle size distribution properly, powder was subjected to ultrasonic deagglomeration for 10 min (further increase in time did not show any improvement) in an ultrasonic bath, followed by particle size distribution characterization (Figure 3). Discarding the obvious agglomerates, D10 = 15.8 μm, D50 = 21.8 μm, and D90 = 28.9.

Before sintering, the powder was compacted in a mold using a hydraulic press at room temperature and a pressure of 7–9 MPa. The sample containing the compacted powder was then placed in a muffle furnace, where it was held for 1 h at the temperatures specified above. The resulting samples for subsequent testing were parallelepiped-shaped with dimensions of a = 8 cm, b = 1 cm, and h = 0.4 cm. The following formula was used to calculate the porosity of the sintered samples:

$$P=\left(1-\frac{\rho_{prac.}}{\rho_{0}}\right)\times 100\%$$

Quantitative measurements were conducted on at least five samples per independent experiment; the relative standard deviation did not exceed 5% across all datasets.

## 3. Results and Discussion

**Figure 4:**
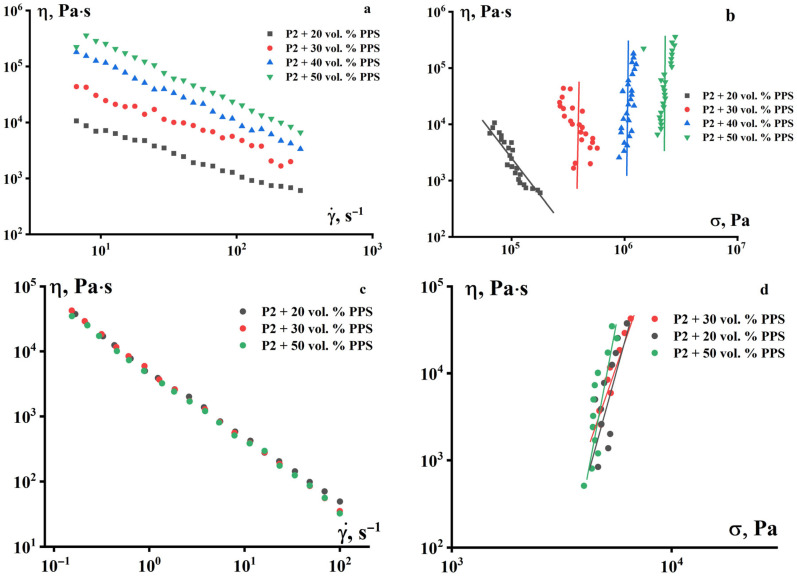
Dependence of the apparent viscosity of suspensions based on PPS and P-2 as a binder on the shear rate (**a**,**c**) and shear stress (**b**,**d**) at two temperatures—50 °C (**a**,**b**) and 120 °C (**c**,**d**).

A study of the viscosity of paraffin- and PPS-based suspensions as a function of shear rate revealed that increasing PPS content leads to a uniform increase in melt viscosity (Figure 4). Figure 4a,c are presented in the traditional manner as a function of apparent viscosity versus shear rate (since the shear rate is typically specified during measurements). Judging from these figures, the suspensions, at first glance, behave like typical non-Newtonian fluids. However, this representation can lead to incorrect conclusions. This is clearly seen in Figure 4b,d, where shear stress, rather than shear rate, is used as the argument.

This analysis reveals that there is no actual flow (except for the 20% suspension at 50 °C), and the constant stress indicates that, during deformation of suspensions containing 30, 40, and 50% PPS powder, the composition slips along the solid wall of the device. Moreover, at low temperatures (data in Figure 4b), the resistance to fluid flow depends on the concentration of the solid phase. Meanwhile, at high temperatures, slip occurs in the paraffin layer regardless of the PPS content (Figure 4d). Interestingly, the sliding velocity increases as stress (acting force) increases. This is reflected by the slope of the straight line in this figure.

**Figure 5:**
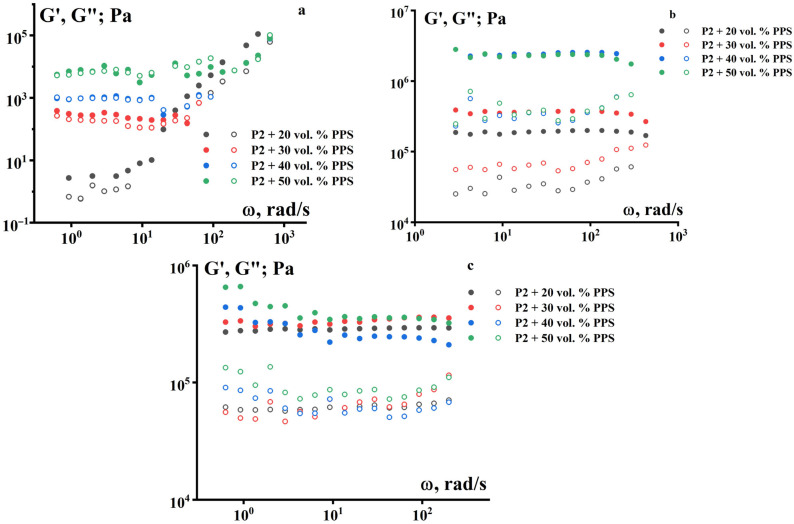
Frequency dependences of the storage modulus G′ (dark) and the loss modulus G″ (light) for suspensions based on PPS with P-2 as a binder at temperatures of (**a**) 80 °C, (**b**) 100 °C, and (**c**) 120 °C.

The frequency dependences of the complex modulus of the suspensions also show that at 80 °C, the storage modulus and loss modulus depend on the frequency only for the composition with 20 vol.% PPS (Figure 5). As the temperature increases, all compositions are characterized by solid-like behavior.

**Figure 6:**
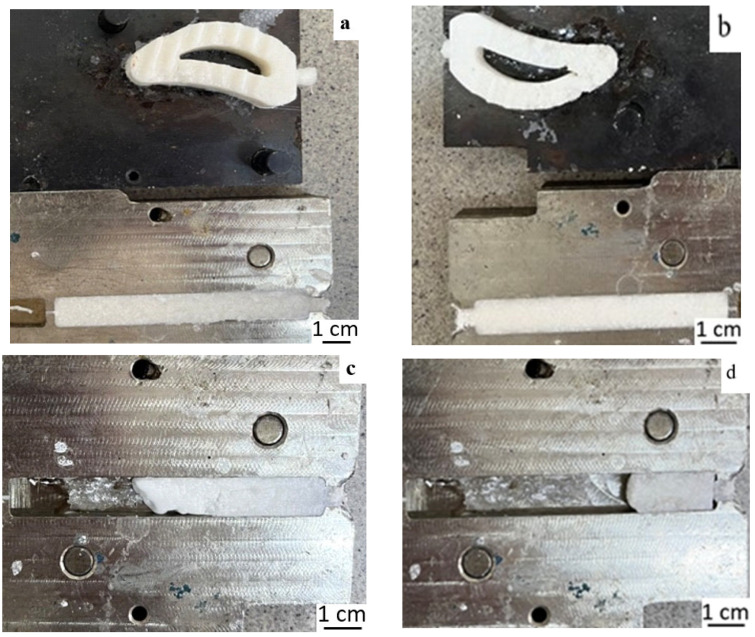
Optical images of PPS samples obtained by casting suspensions with paraffin as a binder with a polymer content of 20 vol.% (**a**), 30 vol.% (**b**), 40 vol.% (**c**), and 50 vol.% (**d**).

The obtained results of the study of the rheological properties of PPS suspensions in paraffin show that they (except for 20 vol.% suspension) exhibit a solid-like behavior, confirmed by the independence of the elastic modulus from frequency. This means that they cannot flow as required for casting. This is confirmed by the behavior of PPS suspensions in paraffin during injection molding. Figure 6 shows that a composition containing 20 vol.% PPS can produce bar samples and complex products of the required shape. However, even with a PPS content of 30 vol.%, the complex product sample produced has significant defects, and suspensions with concentrations of 40 and 50 vol.% PPS fail to fill the mold.

Although 20 vol.% of the composition in paraffin produces satisfactory results when filling the mold, further operations with such a low polymer content cannot produce a high-quality part during sintering. Therefore, paraffin cannot be used as the sole binder for power injection molding of PPS powders.

**Figure 7:**
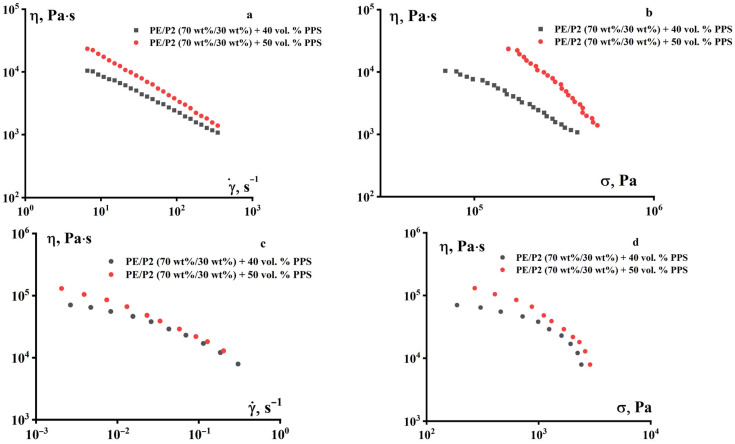
Dependences of the apparent viscosity of suspensions with a mixture of PE and P-2 (70/30) as a binder with different PPS contents on the shear rate (**a**,**c**) and stress (**b**,**d**) at 120 °C (**a**,**b**) and 160 °C (**c**,**d**).

The introduction of PE into the binder composition leads to a significant improvement in the rheological properties of the composition. As can be seen in Figure 7a,b, the compositions with 40 and 50 vol.% PPS flow with the addition of PE and demonstrate non-Newtonian behavior typical of concentrated suspensions with a natural increase in viscosity with an increase in the content of solid filler (PPS powder). Figure 7c,d shows the results of measurements in a lower shear rate range at a temperature of 160 °C.

**Figure 8:**
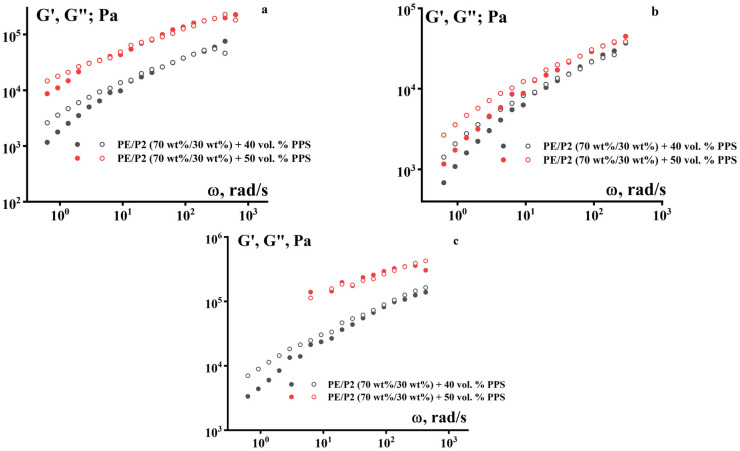
Frequency dependences of the storage modulus G′ (dark) and the loss modulus G″ (light) for PPS suspensions using a PE + P-2 mixture (70/30) as a binder at three temperatures: (**a**) 120 °C, (**b**) 140 °C, and (**c**) 160 °C.

The frequency dependences of the dynamic modulus also exhibit characteristics of viscoelastic materials. A typical increase in the storage modulus (G′) and loss modulus (G″) with increasing frequency is observed (Figure 8). However, it is noteworthy that the values of G′ and G″ are close to each other. This means that irreversible deformations during the formation of the product’s geometric shape are caused not only by flow but also by plastic deformations of the suspensions.

These rheological characteristics enable stable mold filling for both 40% and 50% PPS suspensions in the temperature range of 120–160 °C, which is more than 100 °C lower than the melting point of PPS.

**Figure 9:**
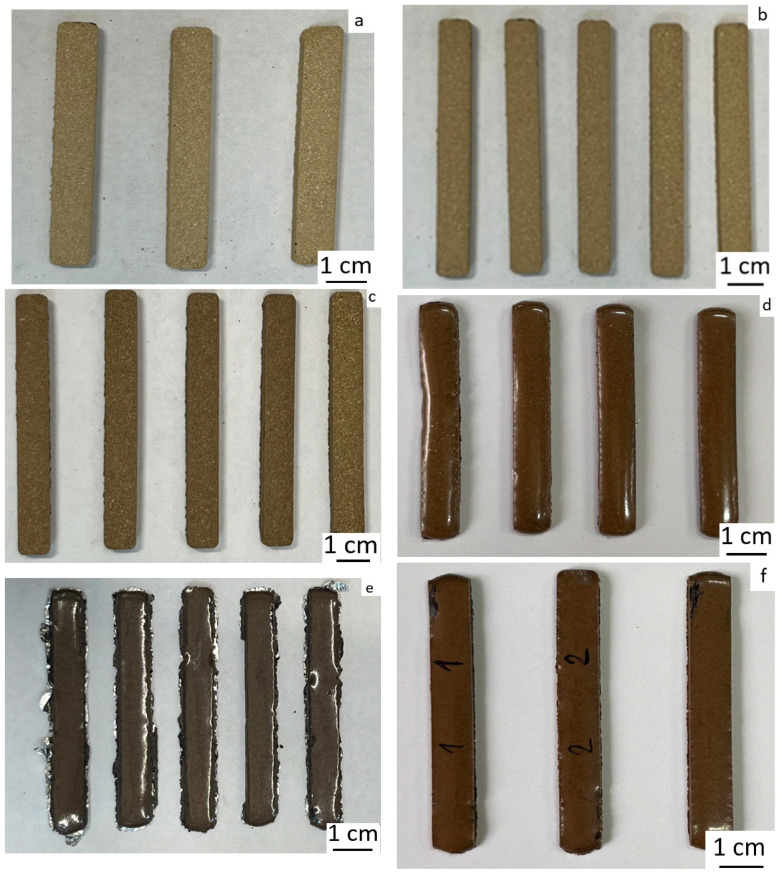
Photographs of PPS samples after sintering: (**a**) 270 °C, (**b**) 280 °C, (**c**) 290 °C, (**d**) 300 °C, (**e**) 310 °C, (**f**) 320 °C.

The sintering of PPS powder is a stage of the technological process that largely determines the quality of the final product. At this stage, due to diffusion processes, the powder consolidates and forms the final product. To determine the optimal sintering conditions for PPS powder, sintering was monitored at various temperatures for 1 h. As shown in Figure 9, up to 290 °C, the samples retain their rectangular shape, but the powder particles within the sample remain intact, not forming a monolith. Upon reaching 300 °C, the particles melt and fuse, forming monolithic products. This is also confirmed by the porosity measurements of the obtained samples. At temperatures below 300 °C, porosity is unacceptably high (above 30%); for samples sintered at 300 °C, porosity drops abruptly to 6.7%.

**Figure 10:**
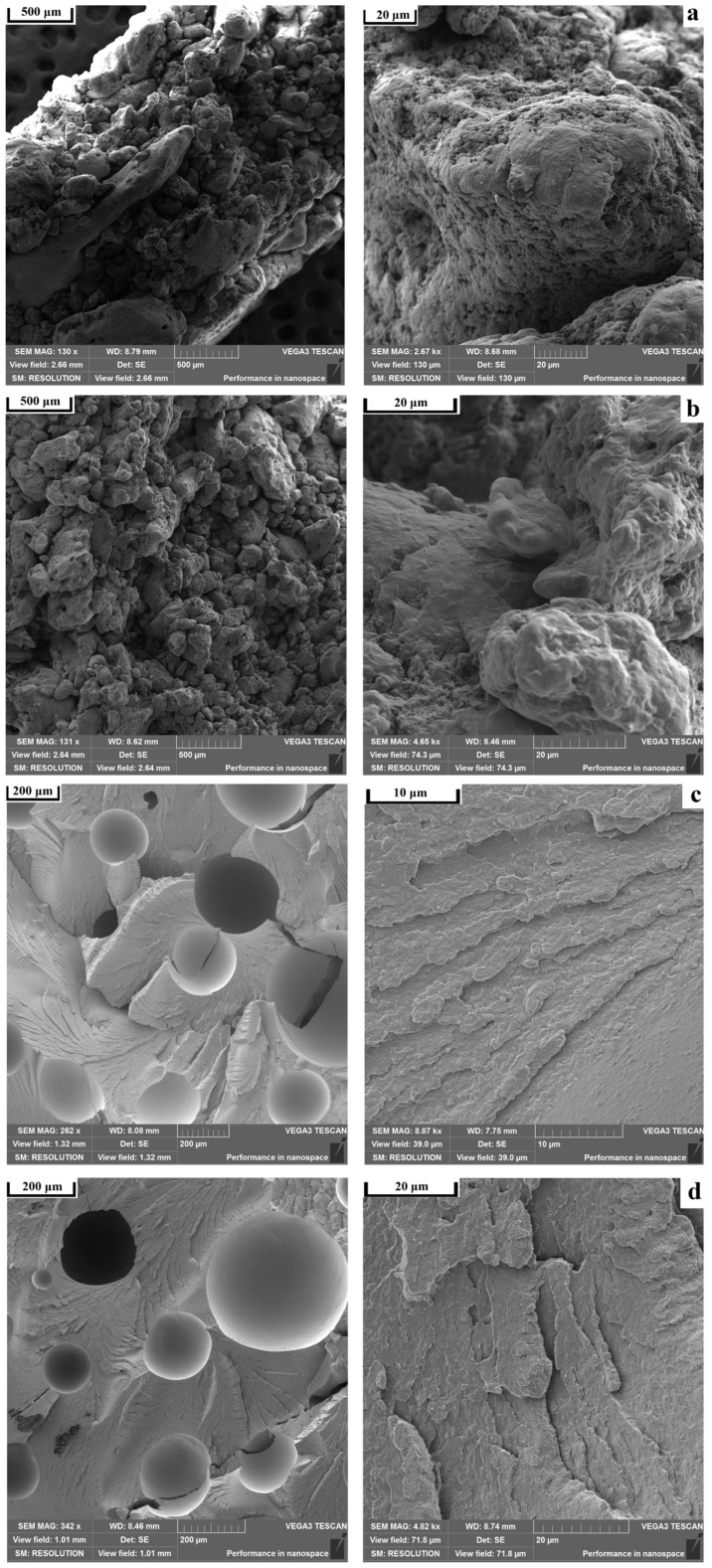
SEM images of PPS samples after sintering at (**a**) 280 °C, (**b**) 290 °C, (**c**) 310 °C, and (**d**) 320 °C at different magnifications.

Figure 10 demonstrates that at sintering temperatures below 300 °C, the samples are characterized by a loose microstructure, in which PPS powder particles sintered together are clearly distinguishable. Upon the elevation of the sintering temperature above 300 °C, a certain quantity of large pores is observed on the fracture surfaces of the samples; however, primary PPS particles are no longer detected due to their melting and consolidation of the polymeric material.

**Figure 11:**
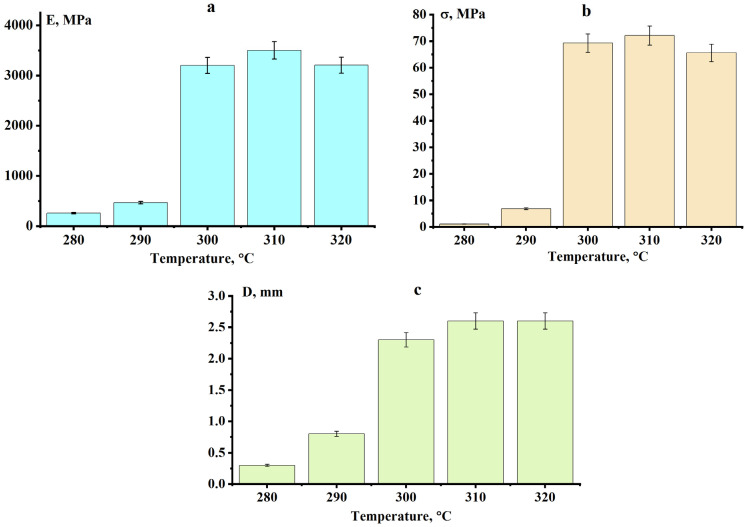
Dependences of the elastic modulus (**a**), tensile strength (**b**), and bending strain (**c**) on the sintering temperature of PPS.

The mechanical properties of the sintered samples are a decisive factor. A study of the mechanical properties showed (Figure 11) that at a sintering temperature of 300 °C, there is a sharp increase in the elastic modulus, tensile strength, and tensile strain. The flexural modulus of the sintered samples reaches the level of cast PPS samples—3200 MPa; however, the flexural strength and strain are somewhat lower (the flexural strength and the breaking strain for the cast samples are 103 MPa and 5.3 mm, respectively). Further increases in sintering temperature have virtually no effect on these properties.

When using high-temperature engineering polymers, composites containing various solid fillers are of interest. Therefore, experiments were conducted using powder injection molding with PPS modified with fillers of various types, widely used in polymeric materials: nanoscale silicon dioxide (aerosil), microsized chalk and talc, and carbon fibers (CF). These experiments included evaluating the rheological and process properties of the composite materials, producing prototypes, and measuring their key physical and mechanical properties.

**Figure 12:**
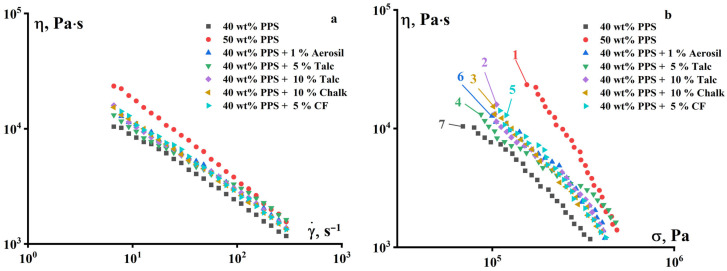
Dependence of the viscosity of suspensions based on a mixture of PE and P-2 as a binder, which contains 40% PPS and various fillers, on the shear rate (**a**) and stress (**b**). T = 120 °C.

Figure 12 shows that fillers lead to a slight increase in viscosity, but they do not affect the flow pattern. All composites with fillers have lower viscosity compared to the samples containing 50 vol.% PPS.

**Figure 13:**
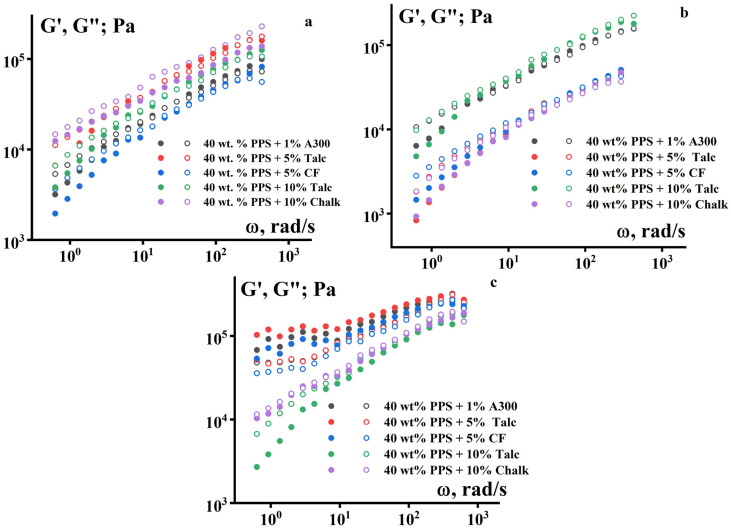
Frequency dependences of the storage modulus G′ (dark) and the loss modulus G″ (light) for suspensions based on a mixture of PE + P-2 (70/30) as a binder and PPS (40 vol.%) with different solid fillers: (**a**) 120 °C, (**b**) 140 °C, (**c**) 160 °C.

At the same time, the introduction of fillers in all cases increases the material’s rigidity (Figure 13), but the materials still behave as viscoelastic bodies, for which G′ and G″ increase with increasing frequency. Notably, at 180 °C, the elastic modulus shows no dependence on frequency, which is typical for solids. This behavior is possibly due to paraffin evaporation, which leads to a significant increase in the proportion of the elastic component of deformation.

**Figure 14:**
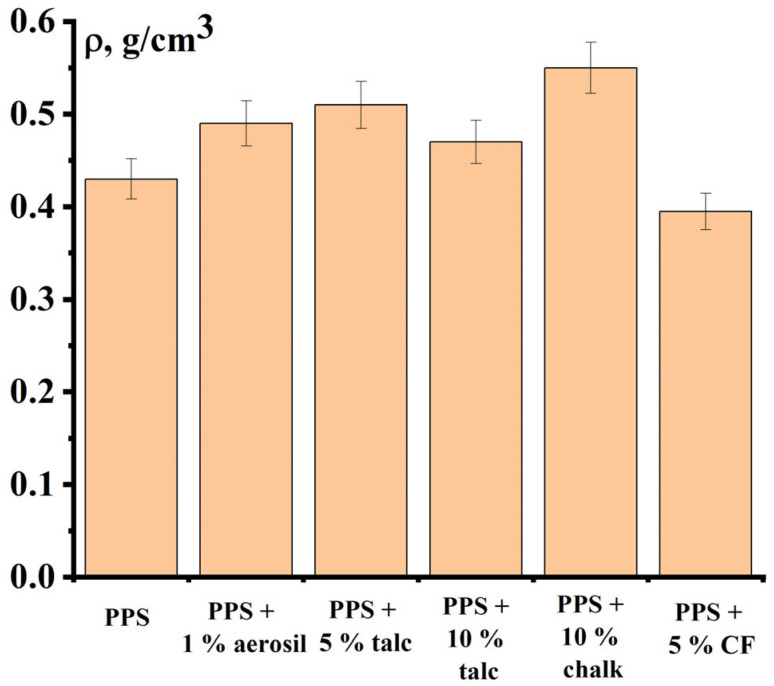
Bulk density of PPS with fillers.

The addition of fillers to the polymer results in a slight increase in the bulk density of the PPS powder (Figure 14). The highest bulk density is observed for the composite powder containing chalk. The exception is CF, which, on the contrary, leads to the loosening of the powder.

**Figure 15:**
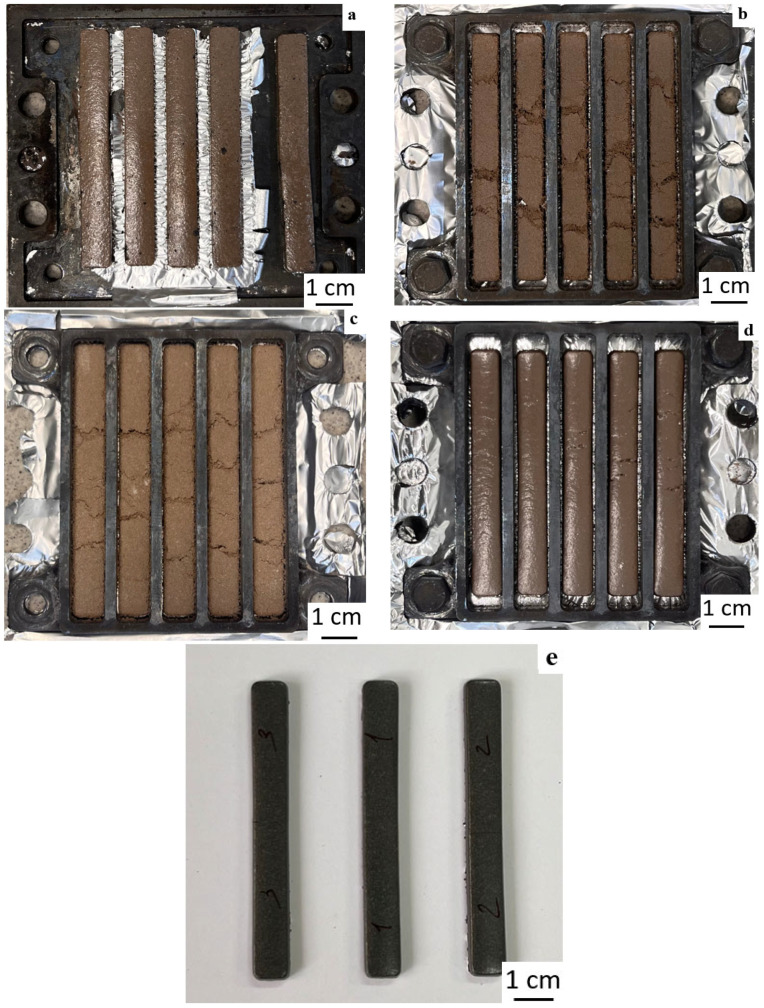
Photographs of PPS samples with filler after sintering: (**a**) PPS + 1 vol.% aerosil, (**b**) PPS + 5 vol.% talc, (**c**) PPS + 10 vol.% talc, (**d**) PPS + 10 vol.% chalk, (**e**) PPS + 5 vol.% CF.

The sintering of the composite powders, as well as pure PPS, was carried out at 300 °C for 1 h. Figure 15 shows that the addition of talc leads to significant defects after sintering. In all other cases, perfectly good samples were obtained. Apparently, talc, due to its hydrophobic nature (wetting angle θ > 90 was measured by a sessile drop method and was proved right, as we were unable to perform measurements using the Washburn method), coats the polymer particles, preventing their fusion. This is also confirmed by the fact that the talc-containing samples have a matte surface, while the samples with other fillers have a glossy surface after fusion.

**Figure 16:**
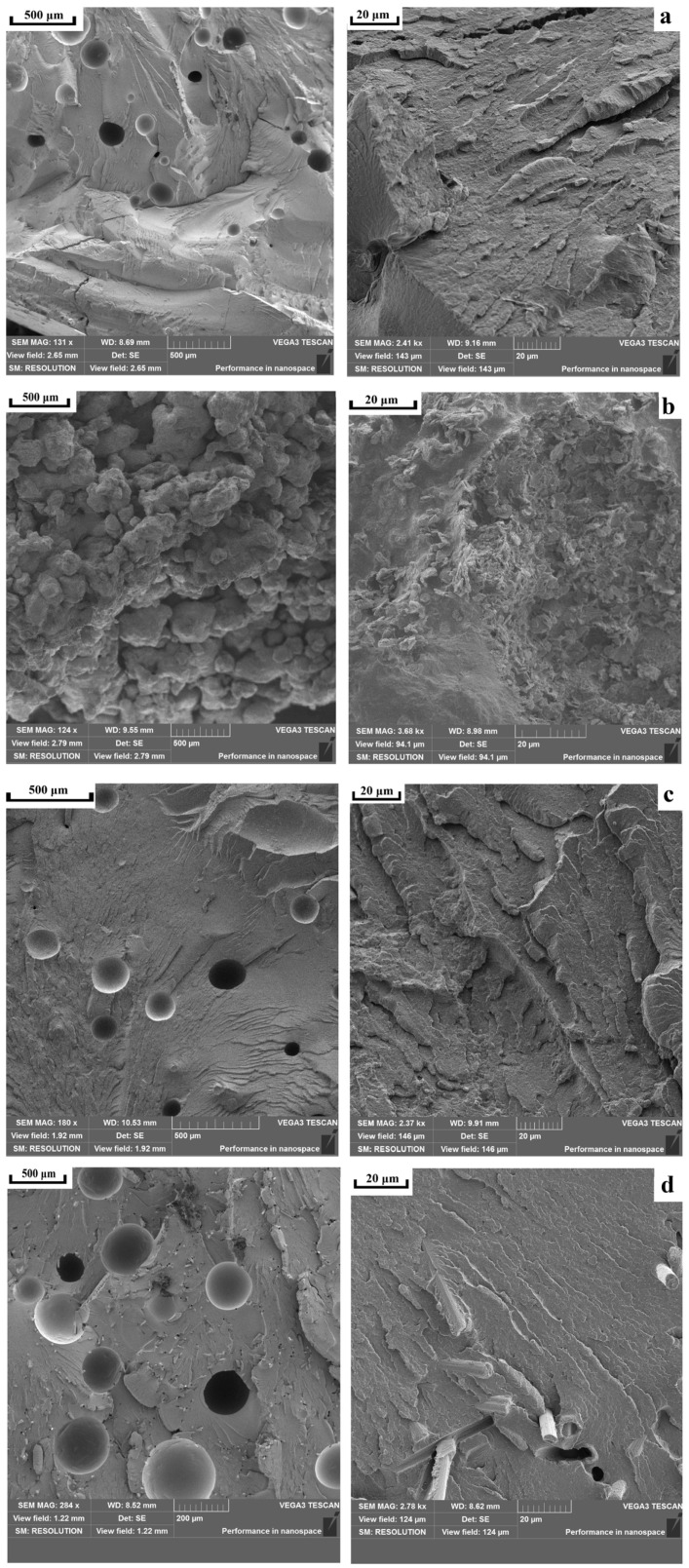
SEM images of PPS samples with filler after sintering: (**a**) PPS + 1% aerosil, (**b**) PPS + 10% talc, (**c**) PPS + 10% chalk, (**d**) PPS + 5% CF.

**Figure 17:**
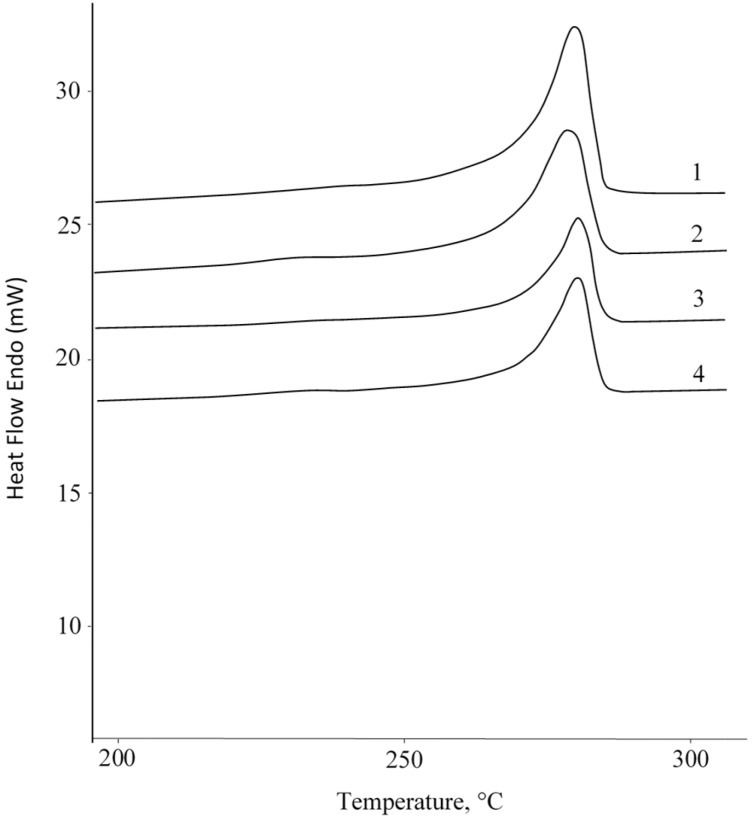
DSC curves: (1) PPS, (2) PPS + 10% chalk, (3) PPS + 10% talc, (4) PPS + 5% CF.

Scanning electron microscopy (Figure 16) shows that sintered samples containing aerosil, chalk, and carbon fibers have large pores on their fracture surfaces, averaging around 200 μm in diameter. Most of the material has melted and formed a dense, well-consolidated structure. The carbon fiber composite (Figure 16d) displays additional, smaller pores caused by fibers being pulled out of the polymer matrix during fracture. The fibers are randomly distributed and lack any preferred direction—a result of the forming process, which does not apply directional pressure. Notably, the polymer matrix adheres well to the carbon fiber surface, with no visible gaps at the interface (marked by circles). The talc-filled sample (Figure 16b) presents a different picture; it shows a loose, undensified structure similar to unfilled samples sintered below 300 °C (Figure 9). At higher magnification, numerous talc particles coat the surface of the PPS particles (shown by white circles). However, thermal analysis (Figure 17) reveals that this composite melts at essentially the same temperature with the same heat absorption as pure PPS and other composites. This finding suggests that the talc particles on the PPS surface actually prevent the particles from fusing together after melting.

**Figure 18:**
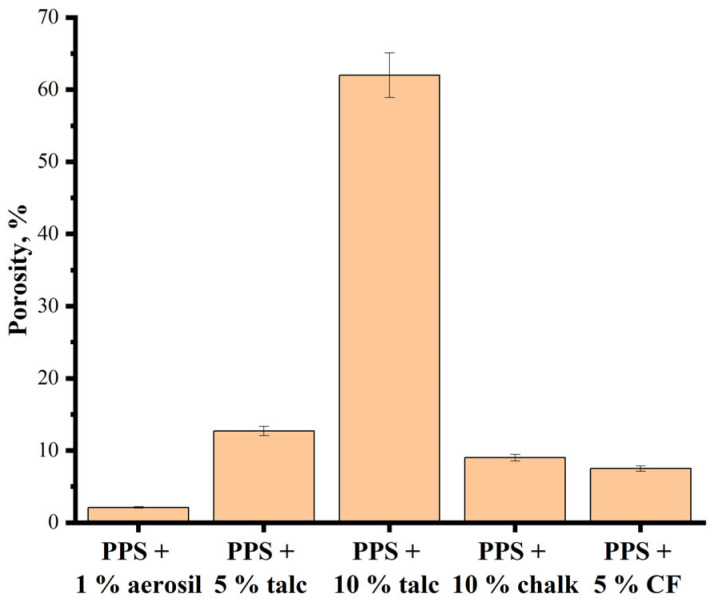
Porosity of sintered at 300 °C samples of PPS with different fillers.

Figure 18 presents the porosity values of sintered PPS samples with various types and contents of fillers. The sample containing aerosil exhibits the lowest porosity. Samples containing chalk and carbon black are characterized by comparable porosity values that do not exceed 10%. The sample with 5% talc demonstrates higher porosity, whereas an increase in talc content to 10% is accompanied by a sharp increase in porosity to 62%. As noted previously, this effect is attributed to the absence of particle coalescence during the sintering process.

**Figure 19:**
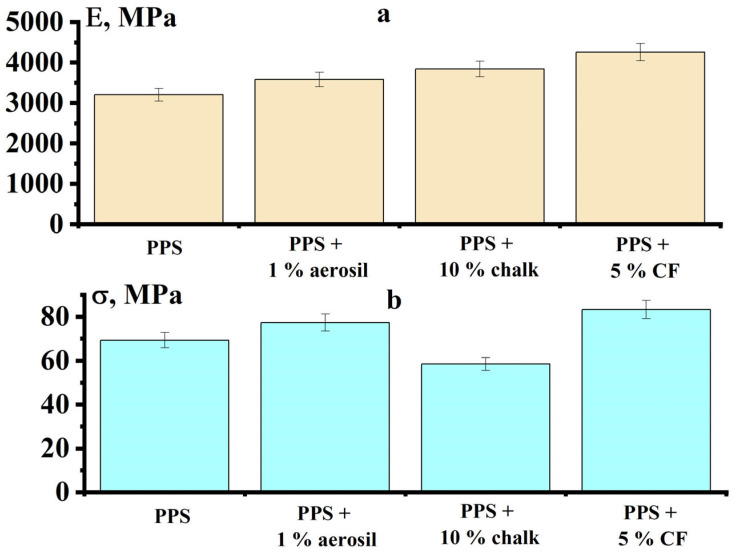
Elastic modulus (**a**) and flexural strength (**b**) of PPS samples containing different fillers.

The addition of solid fillers to PPS leads to a slight increase in the mechanical properties of PPS samples (Figure 19). Samples containing CF demonstrated the highest effect. The elastic modulus and flexural strength increased by 33% and 20%, respectively. Samples containing talc were unsuitable for testing due to defects and a lack of proper sintering.

**Figure 20:**
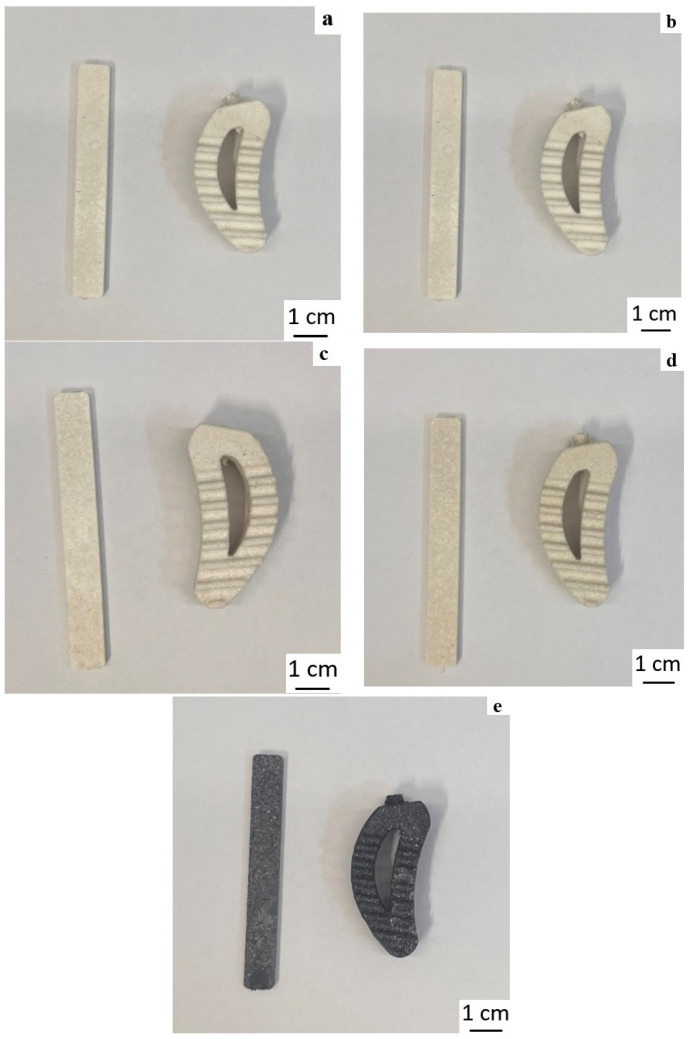
Photos of standard bars (**left**) and complex-shaped parts (**right**) with 40 vol.% PPS in PE-P2 samples (70/30) as a binder with different fillers: (**a**) PPS + 1 vol.% aerosil, (**b**) PPS + 5% vol. talc, (**c**) PPS + 10 vol.% talc, (**d**) PPS + 10 vol.% chalk, (**e**) PPS + 5 vol.% CF.

Thus, all materials with composite powders demonstrated good casting properties. Figure 20 shows samples of standard bars (ISO 294-1:2017 [28]) and complex-shaped parts, which are a prototype of the spinal cage, produced by powder injection molding. It is evident that in all cases, the resulting samples are of good quality, with all geometric elements preserved and free of defects such as underfills, pores, etc.

## 4. Conclusions

A study of the rheological properties of polyphenylene sulfide suspensions and composites with various solid additives allowed the determination of the optimal binder composition for the powder molding of parts from these materials. It was found that pure paraffin cannot be used as a binder, but a 30:70 mixture with polyethylene enables powder molding with a polymer content of at least 40 vol.% in the composite. The optimization of sintering conditions led to the selection of a temperature of 300 °C, with stepwise heating to this temperature. This resulted in the production of high-quality parts, including those with complex geometric configurations, with a strength close to that of cast samples.

## Data Availability

The original contributions presented in this study are included in the article. Further inquiries can be directed to the corresponding author.

## References

[1] Malkin A.Y., Kulichikhin V.G., Mityukov A.V., Kotomin S.V. (2020). Deformation Properties of Concentrated Metal-in-Polymer Suspensions under Superimposed Compression and Shear. Polymers.

[2] Chen X.H., Lu J., Lu L., Lu K. (2005). Tensile Properties of a Nanocrystalline 316L Austenitic Stainless Steel. Scr. Mater..

[3] Ro C.-S., Park J.-N., Jung H.-B. (2017). A Study on the Injection Molding Analysis of the Metal Powder Material. J. Korea Acad. Ind. Coop. Soc..

[4] Cherry J.A., Davies H.M., Mehmood S., Lavery N.P., Brown S.G.R., Sienz J. (2015). Investigation into the Effect of Process Parameters on Microstructural and Physical Properties of 316L Stainless Steel Parts by Selective Laser Melting. Int. J. Adv. Manuf. Technol..

[5] Zhang T., Jiang Z., Wu J., Chen Z. (1990). Influence of Rheological Behavior of Ceramic Mixes on Injection Molding of Ceramic Compacts. J. Am. Ceram. Soc..

[6] Basir A., Sulong A.B., Jamadon N.H., Muhamad N. (2021). Feedstock Properties and Debinding Mechanism of Yttria-Stabilized Zirconia/ Stainless Steel 17-4PH Micro-Components Fabricated via Two-Component Micro-Powder Injection Molding Process. Ceram. Int..

[7] Sommer F., Walcher H., Kern F., Maetzig M., Gadow R. (2014). Influence of Feedstock Preparation on Ceramic Injection Molding and Microstructural Features of Zirconia Toughened Alumina. J. Eur. Ceram. Soc..

[8] Slonov A.L., Zhansitov A.A., Musov K.V., Tlupov A.F., Baykaziev A.E., Rzhevskaya E.V., Khashirova S.Y., Malkin A.Y. (2025). Powder Casting of Polyetheretherketone and Polyphenylene Sulfone: Sintering. Powder Technol..

[9] Khashirova S.Y., Slonov A.L., Zhansitov A.A., Musov K.V., Tlupov A.F., Khashirov A.A., Mityukov A.V., Malkin A.Y. (2024). The Rheology of Polyether Ether Ketone Concentrated Suspensions for Powder Molding and 3D Printing. Polymers.

[10] Wang X., Lin Z., Fu Y., Chen X., Chen Y., Yang Y., Li X., Zeng X., Wen Y., Yang Y. (2025). High-Temperature Resistant Semi-Crystalline Poly(Ether Ether Ketone) Separator. Energy Storage Mater..

[11] Oh J.W., Gal C.W., Shin D., Park J.M., Yang W.S., Park S.J. (2018). Powder Injection Molding Process in Industrial Fields. J. Jpn. Soc. Powder Powder Metall..

[12] Zuo P., Tcharkhtchi A., Shirinbayan M., Fitoussi J., Bakir F. (2019). Overall Investigation of Poly (Phenylene Sulfide) from Synthesis and Process to Applications—A Review. Macromol. Mater. Eng..

[13] Gu J., Guo Y., Yang X., Liang C., Geng W., Tang L., Li N., Zhang Q. (2017). Synergistic Improvement of Thermal Conductivities of Polyphenylene Sulfide Composites Filled with Boron Nitride Hybrid Fillers. Compos. Part A Appl. Sci. Manuf..

[14] Rahate A.S., Nemade K.R., Waghuley S.A. (2013). Polyphenylene Sulfide (PPS): State of the Art and Applications. Rev. Chem. Eng..

[15] Chen G., Mohanty A.K., Misra M. (2021). Progress in Research and Applications of Polyphenylene Sulfide Blends and Composites with Carbons. Compos. B Eng..

[16] Do H.-G., Lee P.-C., Cho B.-G. (2025). Improvement of Bonding Strength Between Polyphenylene Sulfide/Glass Fiber Composites and Epoxy via Atmospheric-Pressure Plasma Surface Treatment. Polymers.

[17] Alturki Y.A., Al Munif E.H., Alarawi A., Banjar H. Chemical Thermal Performance of Nonmetallic Polymers, PEEK, PVDF, PPS and SMPs in Extreme Downhole Conditions. Proceedings of the Middle East Oil, Gas and Geosciences Show (MEOS GEO).

[18] Wan L., Zhou H., Zhou H., Gu J., Wang C., Liao Q., Gao H., Wu J., Huo X. (2025). Recent Advances in Polyphenylene Sulfide-Based Separators for Lithium-Ion Batteries. Polymers.

[19] Suhareva E.M., Pichugin A.M., Konovalov A.P., Popov K.R., Korneev N.A., Tertishnikov I.V., Saveliev E.N., Alykova E.A., Vaniev M.A. (2025). Synthesis and Application of Polyphenylene Sulphides: Problems and Solutions. Plast. Massy.

[20] (2023). Standard Test Method for Melt Flow Rates of Thermoplastics by Extrusion Plastometer.

[21] Department of Health and Human Services Food and Drug Administration (2024). Testing Methods for Detecting and Identifying Asbestos in Talc-Containing Cosmetic Products.

[22] Chang C.-J., Tu Y.-K., Chen P.-C., Yang H.-Y. (2017). Occupational Exposure to Talc Increases the Risk of Lung Cancer: A Meta-Analysis of Occupational Cohort Studies. Can. Respir. J..

[23] Ciocan C., Godono A., Stefanin S., Boffetta P., Pira E., Clari M. (2022). Risk of Mortality from Respiratory Malignant and Non-Malignant Diseases among Talc Miners and Millers: A Systematic Review and Meta-Analysis. Toxics.

[24] Qu C.-L., Yuan L., Yao W.-H., Gao C., Wu J., Gao K., Lei J., Tsou C.-H. (2023). Enhancement of Tensile, Thermal and Barrier Properties of Polyphenylene Sulfide/Carboxylated Graphene Antibacterial Nanocomposites. J. Polym. Res..

[25] Leong Y.W., Ishak Z.A.M., Ariffin A. (2004). Mechanical and Thermal Properties of Talc and Calcium Carbonate Filled Polypropylene Hybrid Composites. J. Appl. Polym. Sci..

[26] Ghanbari L.N., Previte J.P., Wiggins J.S., McNair O.D. (2025). Thermal History Dependent Rheology, Crystallization, and Fusion Joint Strength of Polyphenylene Sulfide/Carbon Fiber Thermoplastic Composites. J. Thermoplast. Compos. Mater..

[27] Zeidler S., Matkovic N., Kößler F., Puchta A., Fleischer J. (2025). Novel Processes for the Production of Continuous Carbon Fiber-Reinforced Thermoplastic Polymers via Additive Manufacturing and Comparisons. Polymers.

[28] (2017). Plastics—Injection Moulding of Test Specimens of Thermoplastic Materials—Part 1: General Principles, and Moulding of Multipurpose and Bar Test Specimens.

